# PEG-based polyurethane bioadhesive for wet and adaptable adhesion to circumcision wounds

**DOI:** 10.1093/rb/rbaf018

**Published:** 2025-03-20

**Authors:** Yaqiang Jiang, Zhaoguo Zhang, Chengkai Xuan, Xuetao Shi

**Affiliations:** School of Materials Science and Engineering, South China University of Technology, Guangzhou 510640, P. R. China; School of Materials Science and Engineering, South China University of Technology, Guangzhou 510640, P. R. China; Guangzhou SoonHeal Medical Technology Co., Ltd, Guangzhou 510000, P. R. China; School of Materials Science and Engineering, South China University of Technology, Guangzhou 510640, P. R. China; National Engineering Research Centre for Tissue Restoration and Reconstruction, South China University of Technology, Guangzhou 510006, P. R. China; Key Laboratory of Biomedical Engineering of Guangdong Province, South China University of Technology, Guangzhou 510006, P. R. China

**Keywords:** circumcision, wet adhesion, surface adaptability, mechanical adaptability, bioadaptability

## Abstract

Effective wound management is critical in post-operative recovery, particularly in sensitive areas such as circumcision. The aim of this study was to design and assess the efficacy of a novel two-component polyurethane (PU) bioadhesive, designated as PU1000S, for its application in advanced wound care within the context of clinical circumcision procedures. The glue-type bioadhesive was fine-tuned to conformally adhere to the moist tissue surfaces. It rapidly absorbed interfacial water and cured within 160 s, ensuring remarkable surface adaptability to wet tissue surfaces. The PU1000S demonstrated superior lap-shear strength, peaking at 55.12 ± 6.88 kPa, along with exceptional durability. These attributes underscored its strong wet adhesion and remarkable resilience at the interface with moist tissues. Owing to its mechanical adaptability to wet skin tissue, the adhesive layer of PU1000S maintained stability under complex loading associated with twisting, folding and significant volumetric deformation, resulting in minimal debonding. In addition, PU1000S was found to significantly accelerate wound healing by promoting re-epithelialization and collagen deposition, confirming its excellent bioadaptability for initial closure and subsequent tissue repair and regeneration following circumcision. The comprehensive results position PU1000S as a promising candidate for advanced wound care in circumcision, offering superior performance in terms of wet adhesion, durability and bioadaptability. Its application could potentially enhance clinical outcomes and elevate patient satisfaction.

## Graphical abstract

**Figure:**
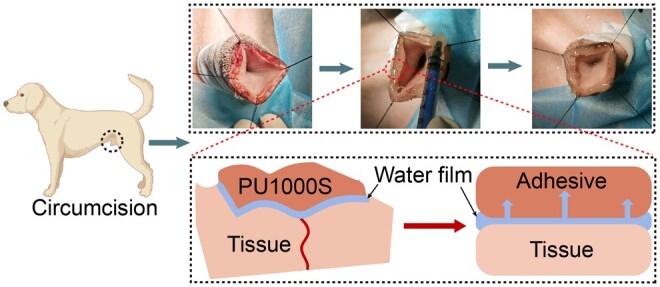

## Introduction

In clinical wound management, sutures and staples have long been recognized as the gold standard for wound closure because of their well-documented effectiveness and reliability [1–3]. However, despite their widespread use, these conventional approaches are not only time-consuming but also associated with a relatively high incidence of complications, such as pain and edema [4, 5]. Particularly for preputial circumcision, traditional suturing and stapling wound closure techniques often require penetration of the delicate and nerve-rich foreskin tissue, which may result in the prolonged post-operative discomfort and poor wound healing. Furthermore, under dynamic conditions such as penile erection, these wound closure techniques often fail to accommodate the natural elasticity and compliance of the foreskin, potentially limiting its natural relaxation and contraction. This mismatch can result in localized stress concentration and pathologic fibrosis surrounding the circumcision wound, even leading to wound dehiscence. The delicate and dynamic nature of the skin requires special consideration in care and treatment of the circumcision wounds. To address these challenges, glue or patch-type bioadhesives have emerged as promising approaches for wound closure, offering a less invasive and more patient-friendly alternative to traditional methods. They are particularly advantageous in scenarios where traditional methods fall short, such as with irregular, hard-to-conform, fragile tissues, as well as wounds with bleeding [6].

Despite their considerable advantages, bioadhesives face several challenges that researchers are actively seeking to overcome. One of the primary issues is ensuring strong adhesion under wet conditions, which is a common scenario in biological tissues. The presence of hydration layers can hinder the intimate contact between the adhesive and the tissue [7, 8], and compete with the adhesive functional groups for interaction [9]. This interference disrupts the formation of robust covalent or noncovalent interactions at the adhesive–tissue interface, leading to a decrease in wet adhesion strength [6, 10]. Additionally, enhancing the flexibility of bioadhesives to better match the mechanical properties of dynamic organs, such as the penis, which undergo significant changes in size and shape during physiological processes, is another challenge. Consequently, there is a need for bioadhesives with an adaptive elastic modulus and stretchability to accommodate the natural movement of tissues, ensuring effective adhesion without compromising the organ's function or comfort [11].

Currently, the predominant strategies to address these challenges are centered around the two primary drainage methods employed by glue-type adhesives: active wetting drainage and swelling drainage [12, 13]. As preformed adhesives, the high water content of conventional patch-type hydrogel adhesives can severely weaken wet adhesion behavior because a weak boundary layer forms on the surface between the hydrogels and tissues [8, 11, 13]. Moreover, patch-type hydrogel adhesives offer a solid matrix and defined geometry, which render them ideally suited for flat tissue wound surfaces but hard-to-conform well to irregularly shaped, hard-to-reach or delicate tissue wounds, such as circumcision wound surface [14, 15]. In comparison, glue-type adhesives, which are liquid in nature, can conformally adhere to the target wet tissue surface, making them particularly effective for treating irregularly shaped and hard-to-reach tissue wounds [13]. As an example of coacervation, nature-inspired fluid colloids are considered ideal platforms for the development of glue-type bioadhesives because of their excellent interfacial active wetting performance in wet environments [16]. However, traditional coacervates have encountered challenges in achieving rapid and stable curing in moist environments, which limits their further application in the field of bioadhesives [12, 17, 18]. The curing kinetics and cohesion mechanism are indeed crucial aspects for the performance of glue-type adhesives. Commercial glue-type bioadhesives, such as cyanoacrylate glue (CA), have been developed with increasing clinical demand. However, CA materials tend to have a high elastic modulus and be brittle after curing, despite their rapid curing kinetics [19], which results in the mismatching of their mechanical properties to those of soft tissues such as foreskins.

**Figure 1.**
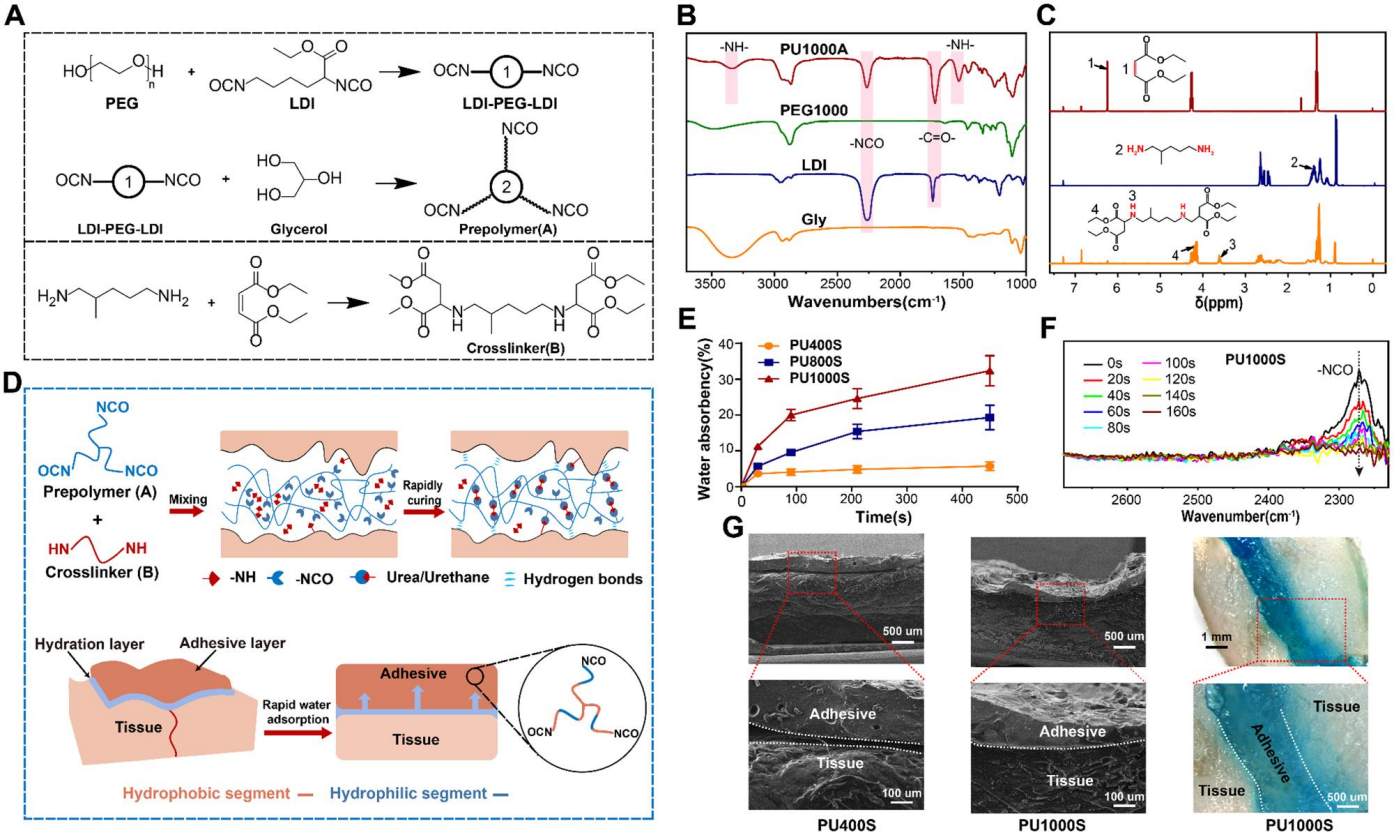
Design and mechanism of wet adhesion for two-component polyurethane bioadhesives. (**A**) Synthesis routes of component A and component B. (**B**) FTIR spectra of the prepolymers. (**C**) ^1^H NMR spectra of the curing agent. (**D**) The wet adhesion mechanism of two-component polyurethane bioadhesives on wet tissue surfaces. (**E**) The instantaneous water absorbency of the PU400S, PU800S and PU1000S adhesives. (**F**) ATR-FTIR analysis of the curing dynamics of PU1000S. (**G**) SEM and stereoscopic images of the adhesion interface of the PU400S and PU1000S adhesives.

On the basis of the above research, we engineered a series of A–B two-component glue-type polyurethane (PU) adhesives with rapid curing and controllable swelling, consisting of a hydrophilic PEG-based prepolymer (Component A) and a small-molecule liquid secondary diamine crosslinker (Component B), as shown in Supplementary Table S1 and Figure 1A. The biocompatibility and molecular designable PU prepolymers, which are polymer melts at room temperature with a low *T*_g_, provide excellent spreadability without the need for water or organic solvents [20–22]. After the two components were mixed, the designed two-component glue-type adhesives were conformally laminated on various tissue surfaces, and the interfacial bonding integrity was maintained via controllable swelling drainage to break down the hydration film. At the same time, the –NH– groups reacted rapidly with the –NCO groups without causing foaming and completed curing within 160 s in a moist environment, which allowed the PU adhesive layer to absorb water rapidly without hindering curing. *In vitro* testing demonstrated that PU1000S achieved strong wet adhesion on moist skin surfaces. Furthermore, the PU tissue adhesive formulation aligns with the mechanical properties of skin tissue and provides the flexibility necessary to accommodate the dynamic conditions typically encountered in biological settings because of its tunable physicochemical and mechanical properties [23]. The *in vivo* experiments conducted on dog prepuce further validated the adaptability of the PU1000S adhesive and its role in promoting circumcision wound healing. This demonstrated the adhesive's potential to provide robust wet adhesion while maintaining the integrity of the tissue interface, even under challenging conditions in the biological environment.

## Materials and methods

### Materials

A series of polyethylene glycols (PEG400, PEG800 and PEG1000) and tetrahydrofuran (THF) were purchased from Sigma–Aldrich. L-Lysine diisocyanate (LDI), anhydrous sodium sulfate (Na_2_SO_4_), diethyl maleate, 2-methyl-1,5-diaminopentane, anhydrous ethanol, glycerol and hematoxylin–eosin were purchased from Shanghai Aladdin Biochemical Technology Co., Ltd. Ether, toluene and hydrochloric acid (HCl) were purchased from Guangdong Reagent Technology Co., Ltd. Ammonium ceric nitrate, dibutylamine, bromophenol blue and PBS buffer were purchased from Shanghai Macklin Biochemical Technology Co., Ltd. Iodophor was purchased from Shandong Lilkang Medical Technology Co., Ltd. Saline solution was purchased from Sichuan Kelun-Biotech Biopharmaceutical Co., Ltd. N-butyl α-cyanoacrylate tissue adhesive (CA) was purchased from Beijing Compont Medical Devices Co., Ltd.

### Synthesis of PU prepolymers (component A)

In the synthesis of PU, the *R* value and OH value are two crucial parameters that dictate the proportion of hydrophilic segments within the polymer and ultimately influence its physical and chemical properties. Drawing upon prior research findings, this study adopted an *R* value of 1.7 and an OH value of 2.5 for the synthesis process. The detailed procedural steps were as follows:

A volume of 50 ml of L-lysine diisocyanate (LDI) solution, with a concentration of 340 mM in tetrahydrofuran, was introduced into a double-necked flask equipped with a condenser immersed in a water bath maintained at 60°C. Via a peristaltic pump, 50 ml of a tetrahydrofuran solution containing 80 mM polyethylene glycol (PEG) was added dropwise to the reaction vessel over time. Following the addition, the mixture was allowed to incubate with gentle stirring. Another peristaltic pump was used to introduce 0.004 M glycerol dropwise into the previously mentioned reaction system. The progression of the reaction was monitored via di-n-butylamine-hydrochloric acid titration. Upon completion of the reaction, the solvent and other small molecules were removed via rotary evaporation. The resulting synthesized PU prepolymer mixture was then sealed airtight and stored in a dry cabinet to preserve its integrity.

### Synthesis of crosslinker (component B)

First, 0.1 mol of diethyl maleate (DEM) and 0.05 mol of 2-methyl-1,5-diaminopentane (MDP) were separately dissolved in anhydrous ethanol. Additionally, 1.5 g of ammonium ceric nitrate was dissolved in water. The solutions of 2-methyl-1,5-diaminopentane in ethanol and ammonium ceric nitrate in water were then combined in a two-necked flask. Using a peristaltic pump, an ethanol solution containing diethyl maleate was gradually introduced into the mixed solution while stirring at room temperature for 24 h. Once the reaction was complete, the mixture was centrifuged at 9000 rpm for 5 min. The supernatant was collected and subjected to extraction with a large volume of diethyl ether. The upper layer of the extract was subsequently concentrated via rotary evaporation to obtain the curing agent used in the two-component PU adhesive.

### FTIR analysis of the PU prepolymers and crosslinker

The series of synthesized PU prepolymers and the crosslinker were characterized via a Fourier transform infrared spectrometer (Nicolet Nexus, Thermo Fisher Scientific, USA). The scanning wavenumber range was set from 4000 to 400 cm^−1^, with a resolution of 4 cm^−1^.

### ^1^H NMR analysis of the crosslinker

The synthesized curing agent was dissolved in CDCl_3_ at a concentration of 10 mg/ml and characterized via nuclear magnetic resonance hydrogen spectroscopy (AVANCE III HD 400, Bruker, Germany) at scan intervals of 16–4 ppm.

### Rheological studies

The rheological characterization of PU prepolymers with varying *R* values and OH values was conducted using a TA Instruments HR20 advanced rotational rheometer. The testing parameters were configured as follows: a 20 mm parallel plate geometry (gap 500 μm) was employed under isothermal conditions at 30°C. Steady-state shear measurements were performed with a linearly ramped shear rate ranging from 0.1 to 100 s^−1^.

### Instantaneous water absorbency and swelling measurement

To accurately simulate real-world conditions, an instantaneous water absorbency measurement was carried out on the adhesive. The A and B components of a two-part PU adhesive were mixed in a 35 mm Petri dish with vigorous stirring to ensure thorough blending. Phosphate-buffered saline (PBS) solution was then added, and the adhesive layer was submerged completely. After a brief immersion period, the PBS was removed, and the difference in mass before (*m*_0_) and after (*m_t_*) soaking, once normalized, provided a measure of the instantaneous water absorption capacity of the two-component PU adhesives via Equation (1):


(1) Water absorbency%=(mt-m0)m0×100%

The PU adhesives were cast into a grooved mold and allowed to cure, resulting in cylindrical samples measuring 10 mm in diameter and 5 mm in height. The initial mass of each sample in its dry state was recorded as *m*_0_. These samples were then immersed in a PBS solution. At regular intervals, the samples were carefully removed, any surface moisture was gently wiped away, and the mass of each sample at a specific time point *t*, denoted *m_t_*, was meticulously measured. The swelling ratios *S* of all the samples were determined via Equation (2):


(2) S=(mt-m0)m0×100%

### Assessment of the curing performance

After rapidly mixing Component A and Component B, the mixture was immediately tested via total internal reflection Fourier transform infrared (ATR-FTIR) spectroscopy. Rapid scanning was performed in the wavenumber range of 400–4000 cm^−1^, with the scanning frequency of once every 20 s. The curing process of a two-component PU adhesive involves a reaction between the –NCO groups present in Component A and the –NH groups within the curing agent. Once the –NCO reactive groups are depleted, the adhesive is deemed fully cured. Consequently, the curing duration of the adhesive can be ascertained by monitoring the alterations in the absorption peaks of these key functional groups via ATR-FTIR.

### Wet adhesion strength assessment

Three distinct methods were employed to test the adhesion strength of PU adhesives on wet pig skin: measuring the lap-shear strength, plane tensile adhesion and interfacial toughness.

#### Lap-shear strength testing

Fresh porcine skins were cut into 50 × 15 mm strips and soaked in PBS buffer solution to maintain a moist state. Two pieces of moist porcine skin were subsequently bonded together with the adhesives with an area of 15 × 15 mm under slight pressure. All the samples were subjected to lap-shear strain at a rate of 50 mm/min until the adhesive joints failed via a universal material testing machine (Intron 5967, Instron, USA) according to the ASTM F2255–05 standard. At least three samples were tested in each group.

#### Plane tensile strength testing

Fresh porcine skins were cut into 15 × 15 mm strips and soaked in PBS buffer solution to maintain a moist state. Two pieces of moist porcine skin were subsequently bonded together with the adhesives with an area of 15 × 15 mm under slight pressure. All the samples were subjected to tensile strain at a rate of 20 mm/min until the adhesive joints failed via a universal material testing machine (Intron 5967, Instron, USA) according to ASTM F2258. At least three samples were tested in each group.

#### Interfacial toughness testing

Fresh porcine skins were cut into 15 × 120 mm strips and soaked in PBS buffer solution to maintain a moist state. Two pieces of moist porcine skin were subsequently bonded together with the adhesives under slight pressure. All the samples were subjected to tensile strain at a rate of 50 mm/min until the adhesive joints failed via a universal material testing machine (Intron 5967, Instron, USA) according to the ASTM F2256 standard. At least three samples were tested in each group.

### Shear adhesion durability assessment

Fresh porcine skins were cut into 10 × 15 mm strips and soaked in PBS buffer solution to maintain a moist state. Two pieces of moist porcine skin were subsequently bonded together with the adhesives with an area of 7 × 10 mm under slight pressure. All adhesive joints were loaded into a dynamic mechanical analysis (DMA Q800, TA, USA) instrument, where cyclic tensile loading at a rate of 200 N/min was employed at the adhesion interface. The tests were conducted at room temperature and involves three stages: (i) the samples were cycled between 0 and 0.5 N for 1000 cycles, (ii) the samples were cycled between 0 and 1 N for another 1000 cycles and (iii) the samples were cycled between 0 and 2 N for the final 1000 cycles. Throughout the test, the force and displacement of the sample were recorded until the adhesive joints were failed. The maximum cyclic shear stresses corresponding to the three stages were 7.14, 14.28 and 28.57 kPa, respectively.

### Analysis of the adhesion interface morphology

Fresh porcine skins were cut into small pieces and soaked in PBS buffer solution to maintain a moist state. PU adhesive was applied to the surface of *ex vivo* porcine skin. Once cured, the adhesive was sectioned through the midpoint to reveal the cross-section. The samples were then freeze-dried and subjected to sputter coating. The adhesion characteristics of the cross-sections were subsequently examined via a scanning electron microscope (MERLIN, Carl Zeiss AG, Germany).

A stereoscopic microscope (Discovery. V12, ZEISS, Germany) was also used to observe the adhesion interface morphology of the wet samples with a stained adhesive layer, as the dehydration process prior to SEM observation can significantly affect the interface morphology.

### Assessment of mechanical properties

#### Tensile testing

The tensile properties of the cured two-component PU adhesives, commercial N-butyl α-cyanoacrylate (CA) tissue adhesive and porcine skins were assessed via a universal material testing machine (Intron 5967, Instron, USA). The adhesives were cured in a customized Teflon mold to obtain strips 50 × 5 × 2 mm in size. The biological tissues were cut into long strips approximately 30 × 10 × 2 mm in size. All the samples were strained at a rate of 50 mm/min until fracture, and at least three samples were tested in each group.

#### Compressive testing

The compressive properties of the two-component PU adhesives and commercial N-butyl α-cyanoacrylate (CA) tissue adhesive were characterized via a universal material testing machine (Intron 5967, Instron, USA). The adhesives were cured in a customized Teflon mold to obtain cylinders with a diameter of 5 mm and height of 4 mm. All the samples were strained at a rate of 5 mm/min until fracture, and at least three samples were tested in each group.

### Dynamic adhesion performance assessment

#### Twist-folding testing

Fresh porcine skins were cut into 50 × 5 mm strips and soaked in PBS buffer solution to maintain a moist state. Two-component PU adhesive and commercial N-butyl α-cyanoacrylate tissue adhesive (CA) were applied to a piece of moist pig skin. Once cured, the skin tissue coated with the adhesive layer underwent twisting and folding to assess whether the adhesive layer could maintain robust adhesion to the wet surface.

#### Expansion testing on a penis-mimicking balloon model

A cylindrical tube was attached to the balloon's inlet, enabling the air to be either inflated into or deflated from the balloon. After the balloon was inflated to a moderate size, two-component PU adhesive and commercial CA were smoothly and evenly applied in a circular pattern on the balloon surface. The maximum diameters of the completely inflated balloons with and without adhesive layer sites were measured. The ratio of the two diameters was recorded as the expansion ratio.

#### Cyclic tensile testing

Two-component PU adhesives and commercial CA were uniformly applied to a piece of moist pig skin and allowed to fully cure, resulting in homogeneous adhesive layers. The samples with adhesive coatings were subjected to cyclic tensile testing via a dynamic mechanical analyzer at ambient temperature, completing 10 cycles of stretching at tensile strains of 50% and 100% at a strain rate of 500%/min.

### Cytotoxicity

Two-component PU adhesives were cured and sterilized by γ-ray irradiation at an intensity of 5 kGy for 30 min. Then, the cured two-component PU adhesives were immersed in basal culture medium (1640 and 12260-014, Gibco, USA) with a 10 mg sample per milliliter of culture medium to obtain the extracts used for biocompatibility evaluations according to guideline document ISO10993-12.

Rat fibroblasts (RFL, 1640, Gibco, USA) were cultured in a carbon dioxide incubator with a 5% CO_2_ atmosphere at 37°C until stable proliferation and normal cellular morphology were observed. The cells were seeded into 48-well plates at a density of 5000 cells per well and cultured with 0.5 ml of extract, with medium changes occurring every two days. The control group was cultured with complete culture medium.

A CCK-8 test (Dojindo Laboratories, USA) was used to evaluate the relative viability of the above samples. The cells were treated with 300 µl of CCK-8 working solution at 1, 4 and 7 days and incubated at 37°C for 45 min. Subsequently, the absorbance values at 450 nm of 100 μl culture supernatant in the 96-well plate were measured with a multifunctional microplate reader.

A live-dead cell staining assay was conducted to evaluate the cytotoxicity of the sample extracts on cells after culturing for 1, 4 and 7 days. A total of 250 μl AM/PI live/dead staining working solution was added to each well, incubated at 37°C for 30 min and observed via confocal laser scanning microscopy (CLSM; Leica TCS SP8, Germany).

### Evaluation of circumcision wound closure

Our research involving three Labrador dogs was approved by the Laboratory Animal Welfare and Ethics Committee (IACUC No. HTSW210620) under Shenzhen Huateng Biopharmaceutical Technology Co., Ltd, ensuring compliance with ethical standards and animal welfare.

Three groups of healthy Labrador experimental dogs were anesthetized, followed by disinfection and hair removal. The prepuces of each dog were subsequently carefully lifted via four hemostatic forceps. A circumferential incision of 1 cm was then made at the end of the dog's penis, and the prepuce tissue was removed with precision via surgical scissors. The circumcision wounds were closed via three different closure methods: PU1000S, commercial N-butyl α-cyanoacrylate (CA) tissue adhesive and traditional suture thread, each applied to the respective groups of experimental dogs. The wound healing process and any signs of infection were monitored and documented by taking photographs on the 8th and 12th days post-surgery.

After the final assessment of the circumcision wound healing process on the 12th day post-surgery, the experimental animals were humanely euthanized. Skin samples extracted from surgical sites were carefully collected and subjected to high-throughput genomic sequencing analysis. This analysis was conducted to assess the expression levels of genes from the IL, TGF, TNF and TLR families, which are pivotal in inflammation. Understanding the expression patterns of these gene families is essential for elucidating the inflammatory dynamics that occur throughout the wound healing process.

Moreover, skin samples were also collected, dehydrated, embedded and sectioned. These sections were then stained with hematoxylin and eosin (H&E) and Masson's trichrome stain and examined via a slide scanning system to assess the wound healing process and tissue regeneration.

### Statistical analysis

The data derived from the tests were repeated at least three times and are expressed as the mean ± standard deviation (SD). *P < 0.05, **P < 0.01 and ***P < 0.001 indicate significant differences between the data. In all cases, differences were considered significant if *P* < 0.05.

## Results and discussion

### Design and characterization of the two-component PU adhesives

The two-component PU adhesive systems were designed as shown in Figure 1A and are detailed in Supplementary Table S1. The *R* value and the OH value are defined as the molar ratio of –NCO/–OH and the average functionality of all polyol components used for synthesis. As previously described, for glue-type bioadhesives, achieving low prepolymer viscosity and a controllable swelling ratio of the adhesive bulk are crucial for effective wet adhesion. Building upon our earlier research [24], we systematically adjusted the *R* value and the OH value to identify a formulation that concurrently possesses low viscosity and controllable swelling properties. The optimal values identified were *R* = 1.7 and OH = 2.5 (Supplementary Table S2 and Figure S3). The successful synthesis of three-arm PEG-based PU prepolymers designated PUnA (component A, Supplementary Table S1 and Figure S1) was verified by the characteristic infrared absorption peaks corresponding to –NCO at 2250 cm^−1^ and –NH– of urethane at 3330 and 1530 cm^−1^ in the FTIR spectrum, as shown in Figure 1B and Supplementary Figure S2A. The resulting prepolymer PU1500A was excluded because of a significant decrease in flow properties. In this study, a liquid secondary diamine was chosen as the crosslinker (component B) and was thoroughly characterized via nuclear magnetic resonance hydrogen spectroscopy (^1^H NMR) (Figure 1C). The complete disappearance of the characteristic peaks at 6.23 and 1.38 ppm (positions 1 and 2), corresponding to the hydrogen atoms on H–C=C–H of diethyl maleate and –NH_2_ in 1,5-diamino-2-methylpentane, clearly indicated the successful synthesis of component B. Furthermore, the molar ratio of hydrogens at position 4 to position 3 in the product was found to be 4.28:1, which is close to the theoretically expected ratio of 4:1, further validating the successful synthesis of component B. Meanwhile, the analysis of the infrared spectrum of component B (Supplementary Figure S2B) showed the complete disappearance of the –C=C– peak at 1639 cm^−1^. This result confirmed the reliability of the above analysis. In the case of tissue adhesives applied to injuries such as skin wounds, a hydration layer inevitably forms on the tissue surface due to the presence of bodily fluids and blood. In this research, strong hygroscopic PU bioadhesives were fabricated by blending hydrophilic prepolymers (component A) and an aliphatic crosslinker (component B), which can rapidly absorb interfacial water at the tissue surface for strong wet adhesion, as illustrated in Figure 1D. The efficient interfacial water absorption capacity of PU adhesives, referred to as PU400S, PU800S and PU1000S adhesives, was enhanced by fine-tuning the molecular weight of PEG (Supplementary Table S1). As shown in Figure 1E, the PU adhesives demonstrated a remarkable increase in their instantaneous water absorbency, which correlated positively with the molecular weight of the PEG. This capability culminated with PEG1000, which has the exceptional ability to absorb 20 wt.% water within a remarkably short span of just 210 s. In addition, the curing time of the glue-type PU1000S was evaluated by tracking the attenuation of the characteristic absorption peaks corresponding to the –NCO groups at 2250 cm^−1^ in the ATR-FTIR spectrum since the proper curing time was crucial for achieving both the desired spreadability and cohesion [25, 26]. As shown in Figure 1F, the PU adhesives of PU1000S achieved complete curing within 160 s due to the high reactivity between the –NH and –NCO groups, which was significantly shorter than the available PU bioadhesives documented in the literature [27–29] and provided sufficient time for the reapplication and adjustment of the adhesive. Furthermore, component B functions as a reactive solvent, keeping the viscosity of the two-component mixture low prior to full curing, which is crucial for ensuring the adhesive's spreadability performance.

When the PU adhesives were applied to moist pig skin, SEM and stereoimages revealed that the PU1000S adhesive mixture maintained more conformal and closer contact with the target tissue than did PU400S (Figure 1G). The water-absorbing PU1000S adhesive broke down the interfacial water layer and adhered to the tissue via the establishment of urea or urethane linkages and hydrogen bonds (Figure 1D), thereby exhibiting remarkable surface adaptability. Further analysis through SEM imaging demonstrated that the PU1000S adhesive layer, when in contact with moist porcine skin, displayed a compact and nonporous structure. This observation suggested that the highly reactive –NH groups within the curing agent efficiently inhibited the foaming reaction between the –NCO groups and water during swift water absorption by the adhesive. Moreover, the integration of hydrophobic curing agents into the PU network markedly constrained excessive swelling by 95.3% (Supplementary Figure S3B) through the aggregation of hydrophobic segments, thereby increasing the PU network density and maintaining the strong cohesive strength of the adhesive layer.

### Wet adhesion strength and shear adhesion durability

**Figure 2.**
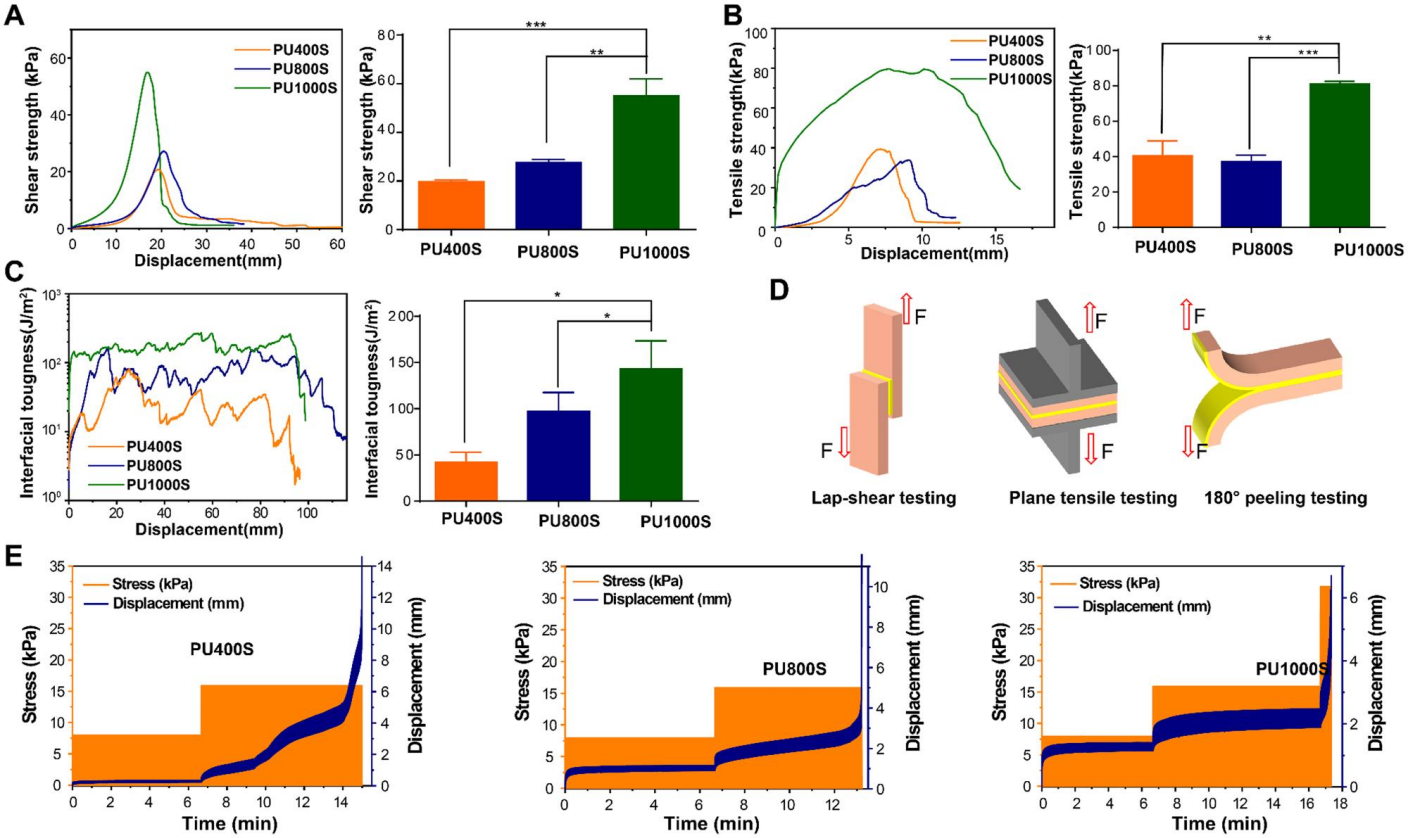
Wet adhesion performance of two-component polyurethane bioadhesives. (**A**) The shear adhesion strength of the PU400S, PU800S and PU1000S adhesives on wet skin tissue. (**B**) Tensile adhesion strength in plane on wet skin tissue for adhesives PU400S, PU800S and PU1000S. (**C**) The adhesion work of the adhesives PU400S, PU800S and PU1000S, as determined by 180° peel tests conducted on wet skin tissue. (**D**) Three distinct mechanical testing methodologies for the assessment of two-component polyurethane bioadhesives. (**E**) The durability of shear adhesion on wet skin tissue for the polyurethane adhesives PU400S, PU800S and PU1000S.

The wet adhesion performance of the two-component adhesive system was evaluated on moist porcine skin tissue models via three distinct mechanical testing methods (Figure 2D). We initially measured the PU adhesive systems on wet porcine skin (Figure 2A–C). As depicted in Figure 2A–C, both the shear strength and tensile strength increased with increasing chain length of the PEG soft segment. PU1000S exhibited the highest lap-shear strength and tensile strength, with values of 55.12 ± 6.88 and 80.96 ± 1.60 kPa, respectively. In terms of interfacial toughness (Figure 2C), PU1000S had the greatest adhesion work, with 143.1 ± 30.39 J/m^2^, compared with PU400S (41.84 ± 11.29 J/m^2^) and PU800S (96.93 ± 20.73 J/m^2^). The superior adhesion performance of PU1000S was attributed to the high water absorbency and flexibility of the molecular chains of PEG1000. These properties effectively removed the hydration layers present on wet skin (Figure 1D).

The shear adhesion durability of the PU adhesives was determined via DMA, as shown in Supplementary Figure S4. Three cyclic shear stresses were sequentially applied to the lap-shear skin joint models: 7.14, 14.28 kPa and 28.57 kPa, with each stress level undergoing 1000 cycles of loading and unloading. At a shear stress of 7.14 kPa, all the PU400S, PU800S and PU1000S groups maintained stable shear adhesion after the initial 1000 cycles of loading–unloading without any significant increase in displacement (Figure 2E). However, once the shear stress was increased to 14.28 kPa, PU400S presented significant shear adhesion degradation, which was verified by the displacement changes. Under shear stress conditions, the shear adhesion of PU800S also exhibited a gradual decline, whereas PU1000S demonstrated remarkable stability until the process reached 28.57 kPa. We believe that PU1000S, formulated with long-chain PEG, offers not only strong wet adhesion but also shear adhesion durability. This suggests that PU1000S is particularly well suited for applications where sustained or fluctuating stress conditions are anticipated.

### Adhesion performance mechanical adaptability

**Figure 3.**
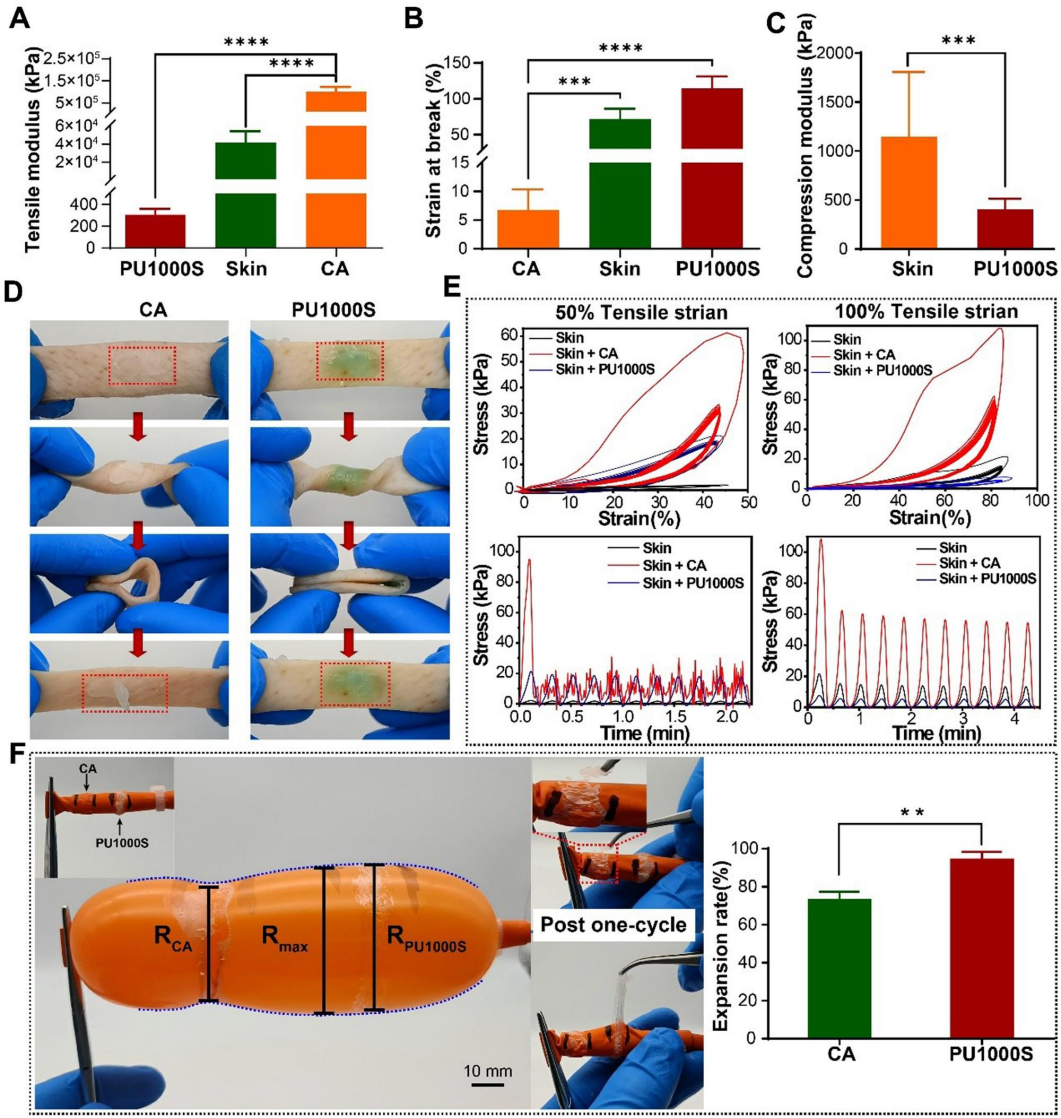
Mechanical adaptability in terms of adhesion performance. (**A**) Tensile moduli of PU1000S, N-butyl α-cyanoacrylate (CA) tissue adhesive and porcine skin. (**B**) Tensile elongation of PU1000S, CA and porcine skin. (**C**) Compression moduli of PU1000S and porcine skin. (**D**) Twisting and folding test of the PU1000S and CA adhesive. (**E**) Assessment of the stability and compliance of the PU1000S and CA adhesives under cyclic tensile testing at 50% and 100% elongation on wet skin surfaces; the adhesive is applied evenly to one side of a piece of wet skin and then subjected to tensile tests with the skin. (**F**) Process of expansion testing in a penis-mimicking balloon model and comparison of the radial expansion ratios for the PU1000S and CA adhesives.

The foreskin is an elastic layer of skin that covers the glans of the penis. It stretches in real time to adapt to the penis's increased volume and length during an erection; this elasticity and ductility are important physical properties that fulfill crucial physiological functions. Tissue adhesives designed for these areas, such as circumcision, must possess a low elastic modulus or good stretchability to adapt to the complex mechanical environment [30–32]. As shown in Figure 3A and Supplementary Figure S5A and B, the significantly greater elastic modulus of the N-butyl α-cyanoacrylate tissue adhesive (CA) at 1.01 ± 0.22 GPa than that of porcine skin (41.49 ± 12.64 MPa) indicated a high degree of elastic modulus mismatch [33]. This disparity severely constrained the skin's capacity for twisting and folding, rendering it impossible to achieve a 180°-fold within the adhesion area (Figure 3D and Supplementary Movie S1). After just one cycle of twisting and folding, the CA layer demonstrated pronounced adhesion failure. Conversely, PU1000S presented a lower tensile modulus (305.60 ± 55.16 kPa) and remarkable stretchability (114.40 ± 16.92%) than did skin (Figure 3A and B and Supplementary Figure S5A and B). The same compressive modulus result (450.3 ± 108.3 kPa) was also observed in Figure 3C and Supplementary Figure S5C compared with that of pig skin (1148 ± 660.4 kPa). The PU1000S adhesive layer was resistant to the complex loading associated with twisting and folding and exhibited no significant debonding because it mechanically conforms to wet skin tissue [34] (Figure 3D).

The adaptability was further evaluated over a more extended strain range. Considerable cyclic loading up to 50% and 100% tensile strains was applied to assess the stretchability compliance [35] of PU1000S (Figure 3E). The CA group always required higher levels of stress under the same strain, and the adhesive bulk was already destroyed in the initial cycle. In contrast, the PU1000S group maintained a consistently lower and stable stress value throughout the entire series of 10 cycles, indicating minimal constraints on extensive tensile stretching. To mimic the physiological changes between erection and flaccidity of the penis following circumcision, CA glue and PU1000S were uniformly applied in a fully circular pattern around a penis-mimicking balloon model, which was then inflated (Figure 3F). During the inflation process, the CA adhesive layer considerably limited the balloon expansion, resulting in an expansion ratio of 73.66 ± 3.687%, which was significantly lower than the 94.91 ± 3.501% for PU1000S. Moreover, throughout the inflation of the balloon model, PU1000S demonstrated an immediate response to the volumetric strain both radially and latitudinally, with changes in the adhesion surface occurring simultaneously with the volumetric strain. The instantaneous response of PU1000S to volumetric strain in both axes underscored the exceptional ductility of PU1000S. As the adhesive experiences extensive strain alongside tissues, ductility provides an effective way to evenly spread mechanical stress across the adhesion area, significantly reducing stress concentration [36, 37]. This mechanical adaptability helped prevent abrupt delamination or failure of the adhesive layer, a common issue with CA glue, as shown in Supplementary Movie S2, thus enhancing the adhesive's stability and reliability. These experiments demonstrated that PU1000S readily adapted to significant tensile and volumetric deformations in multiple directions, suggesting that it could conform well to the dynamic volumetric changes in the penis.

### *In vivo* circumcision wounds adhesion evaluation

It is crucial to assess the biocompatibility of adhesives before *in vivo* application. As shown in Supplementary Figure S6, the relative viability, cell density and morphology of the cells in the PU1000S group were not significantly different from those in the control group, indicating excellent biocompatibility for wound closure.

**Figure 4.**
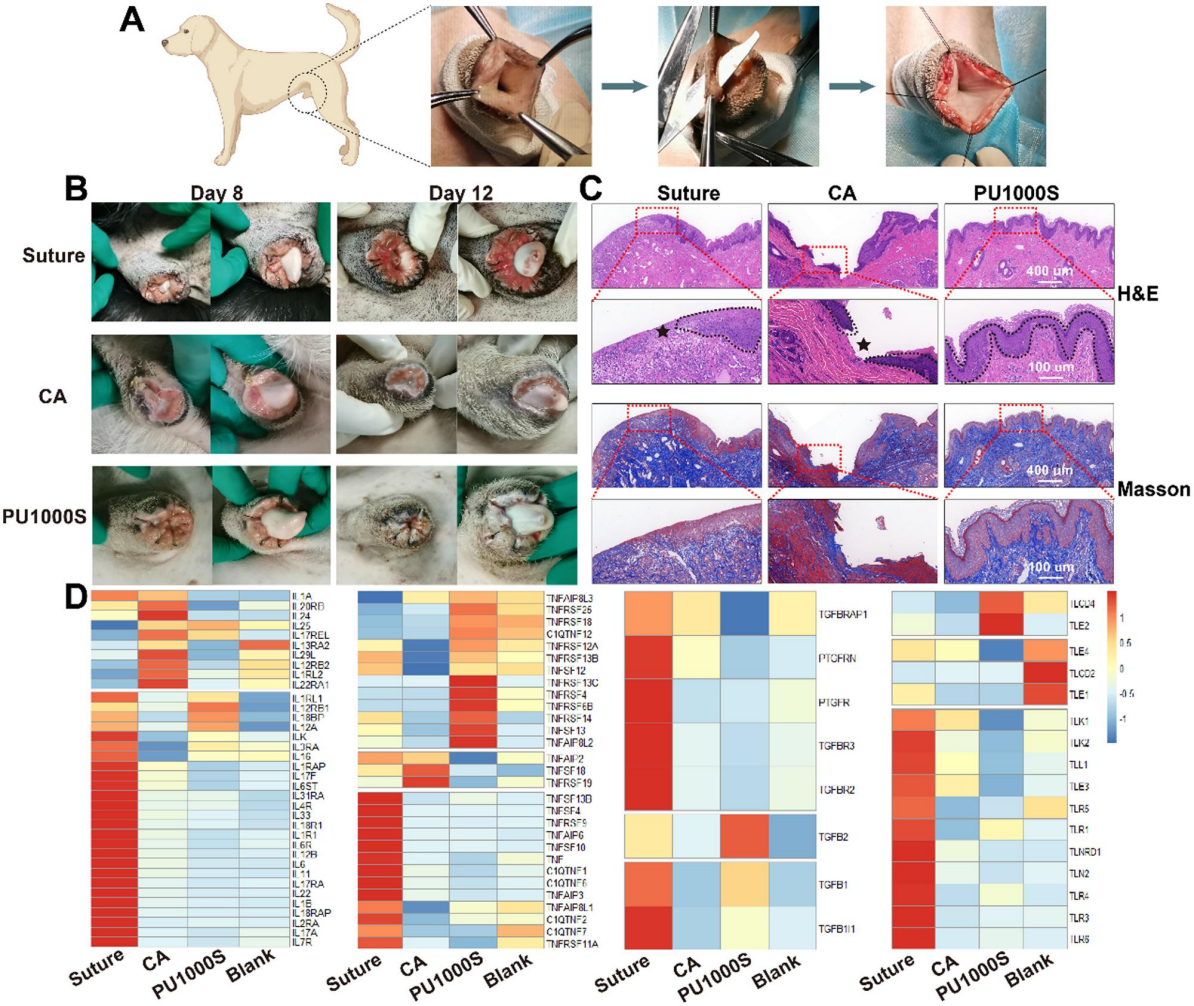
Wound management during circumcision procedures in a model of a laboratory experimental dog. (**A**) Development of an animal model for the study of circumcision. (**B**) Digital images of the results of the healing process after circumcision on days 8 and 12 in the suture, N-butyl α-cyanoacrylate tissue adhesive (CA) and PU1000S groups. (**C**) Images of H&E staining and Masson's trichrome staining on day 12 after surgery in the suture, CA and PU1000S groups; the black dotted lines represent neo-epithelial tissue, and the black pentagrams represent incompletely re-epithelialized wound gaps. (**D**) Heatmap of inflammation-related genes on day 12 after surgery in the suture, CA and PU1000S groups.

Three laboratory experimental dog models for penile circumcision surgery were successfully established. As depicted in Figure 4A, the circumcision wounds exhibited a complex morphology before treatment, accompanied by bleeding. Photographs depicting the wound modeling and closure process in the circumcision of the suture, N-butyl α-cyanoacrylate tissue adhesive (CA) and PU1000S groups are presented in Supplementary Figure S7. In the suturing group, a substantial amount of bleeding occurred because the suture threads pierced the foreskin tissue, resulting in secondary damage. In comparison, the application of CA and PU1000S to the circumcision incision was markedly simplified, utilizing a pipette from the commercial CA kit and a standard syringe for PU1000S, respectively. These glue-type adhesives effectively avoid the tissue damage typically associated with sutures. However, the low viscosity of CA makes it challenging to manage the adhesion area precisely. The CA glue sometimes seeps into the inner layer of the foreskin, posing a risk of adhesion between the penis and the foreskin. Moreover, the high elastic modulus of the CA adhesive layer severely restricted the deformation ability of the foreskin, as shown in Supplementary Movie S3. In normal physiological activities, especially during erections, the foreskin needs to have considerable ductility to adapt to the significant volumetric strain of the penis. This mismatch in mechanical properties may cause significant discomfort at the wound site and may ultimately result in adhesive failure. When the results came to PU1000S, upon immediate application, PU1000S conformally covered the circular wound surface and swiftly formed a physical barrier that effectively achieved hemostasis without any loss of PU1000S to the inner layer of the foreskin. The shape-adaptive adhesive layer of PU1000S did not debond and impeded penile erection, as tension forces were exerted on the circular wounds, as depicted in Supplementary Movie S4. Compared with CA, the low elastic modulus and high stretchability allowed PU1000S to adapt mechanically to the circumcision penis erections process [38], as shown in Supplementary Movie S5.

Digital images of the circumcision wound healing process in the suture, CA and PU1000S groups on the 8th and 12th days are presented in Figure 4B. By the 12th day after surgery, the surface of the wounds in the suture group remained bright red, indicating prolonged inflammation, likely as a result of continuous exposure to nonsterile conditions. The expression levels of genes encoding IL-1β, TGF-β and TNF-α indicated that a significant inflammatory response occurred (Figure 4D). In the CA group, a significant amount of purulent discharge seeped from the wound on the 8th day following application. Typically, in the normal wound healing cascade, purulent discharge levels are expected to decrease within a few days. However, the presence of a substantial amount of exudate in the CA group suggested that inflammation was ongoing and that the wound had yet to advance into the proliferative phase of the healing process. By the 12th day, the wound sites in the CA group, which were less intensely red than those in the suture group, remained reddened. This finding suggested that complete wound healing had not been achieved at this time point, and mild inflammatory signs were still evident, which could be verified by the elevated expression levels of IL family genes such as IL-1α, IL-24 and IL-25 on the 12th day (Figure 4D). However, by the 12th day, the wounds in the PU1000S group had significantly regained their normal skin color, with virtually no inflammation-related genes showing high expression levels (Figure 4D).

Further histological assessments, including H&E staining and Masson's trichrome staining, were conducted to comprehensively evaluate the outcomes of wound healing on the 12th day (Figure 4C). H&E staining revealed that the PU1000S group presented a more complete and continuous neo-epidermal layer, whereas the foreskin tissues in the suture and CA groups presented significant wound gaps with incomplete re-epithelialization on the 12th day, as indicated by the black pentagram in Figure 4C. H&E staining revealed that re-epithelialization in the PU1000S group was significantly accelerated compared with that in the suture and CA groups. Re-epithelialization is an essential phase in the wound healing cascade, which results in complete wound closure and serves as a protective barrier against the risk of dehydration and infection at the wound site [39, 40]. In addition, Masson's staining revealed more structured collagen fiber formation around the circumcision wound healing area in the PU1000S group. The enhanced collagen deposition suggested a more organized and efficient wound healing process [41, 42]. All the results showed that the PU1000S adhesive markedly accelerated wound healing by promoting re-epithelialization and collagen deposition, confirming its excellent bioadaptability during initial closure and subsequent tissue repair and regeneration post-circumcision.

## Conclusion

In this study, a range of two-component PU bioadhesives with diverse water absorbency abilities were prepared by modulating the chain length of the hydrophilic soft segment PEG during adhesive synthesis. Intimate wet adhesion between bioadhesives and tissues was observed because the hydration layer at the wet tissue interface was completely eliminated. On the basis of the unique characteristics of the application scenario, we optimized and selected formulations that ultimately identified PU1000S, which exhibited strong wet adhesion to skin tissue (55.12 ± 6.88 kPa). The elastic modulus of PU1000S was 305.60 ± 55.16 kPa, which was lower than the elastic modulus of skin tissue. These findings indicated that the two-component PU adhesive had good adaptability to the skin. In particular, PU1000S maintained excellent adhesion even when subjected to dynamic stress changes such as twisting, folding, expansion and cyclic stretching along with the adhered tissue, demonstrating its ability to adapt well to the dynamic stress environments of penile foreskin areas.

A laboratory experimental dog foreskin circumcision model was used to assess the *in vivo* wound repair performance of PU1000S. Compared with the control group, the PU1000S group demonstrated good adaptation to the circular wound surface and dynamic volumetric changes in the penis, resulting in reduced inflammation and superior healing outcomes. All these findings underscore the significant potential of PU1000S as a promising candidate for advanced wound care, particularly in the context of circumcision clinical settings.

## Supplementary Material

rbaf018_Supplementary_Data
