# Supplementary material for: PEG-based polyurethane bioadhesive for wet and adaptable adhesion to circumcision wounds
Source: Regen Biomater. 2025 Mar 20;12:rbaf018. doi: 10.1093/rb/rbaf018 (PMC12077817; doi:10.1093/rb/rbaf018)
Supplement: rbaf018_Supplementary_Data [file rbaf018_supplementary_data.zip › SI of PU1000S-Finally 25.3.14.docx]

**PEG-based polyurethane bioadhesive for wet and adaptable adhesion to circumcision wounds**

Yaqiang Jiang^a,1^, Zhaoguo Zhang^a,1^, Chengkai Xuan^d^ and Xuetao Shi^a,b,c^*

^a^ School of Materials Science and Engineering, South China University of Technology, Guangzhou 510640, P. R. China.

^b^ National Engineering Research Centre for Tissue Restoration and Reconstruction, South China University of Technology, Guangzhou 510006, P. R. China

^c^ Key Laboratory of Biomedical Engineering of Guangdong Province, South China University of Technology, Guangzhou 510006, P. R. China

^d^ Guangzhou SoonHeal Medical Technology Co., Ltd., Guangzhou 510000, P. R. China

^1^ These authors contributed equally to this work.

*Correspondence address. School of Materials Science and Engineering, South China University of Technology, Guangzhou 510640, P. R. China. Tel: 020-39380267; Fax: 020-39380267; E-mail: [shxt@scut.edu.cn](mailto:shxt@scut.edu.cn)

Supplementary Tables 1-2

Supplementary Figures 1-7

Supplementary Movies S1-S5

**Supplementary Table 1** Synthesis scheme of 4 different polyurethane prepolymers (Component A)

| PUnA | R | OH | PEGn (mol) | LDI (mol) | Glycerol (mol) |
| --- | --- | --- | --- | --- | --- |
| PU400A | 1.7 | 2.5 | PEG400/0.004 | 0.017 | 0.004 |
| PU800A | 1.7 | 2.5 | PEG800/0.004 | 0.017 | 0.004 |
| PU1000A | 1.7 | 2.5 | PEG1000/0.004 | 0.017 | 0.004 |
| PU1500A | 1.7 | 2.5 | PEG1500/0.004 | 0.017 | 0.004 |

**Note: PUnA** is defined as a prepolymer synthesized from PEG with different molecular weights (MWs) (n=400, 800, 1000 or 1500 Dal), whereas **PUnS** refers to a polyurethane adhesive with a corresponding molecular weight (PU400S, PU800S, PU1000S or PU1500S).

**Supplementary Table 2** Synthesis of polyurethane prepolymers at varying R and OH values

| R | OH | PEG_1000_ (mmol) | LDI (mmol) | Glycerol (mmol) |
| --- | --- | --- | --- | --- |
| 2.0 | **2.0** | 4.0 | 8.0 | 0.0 |
| 1.9 | **2.0** | 4.0 | 8.0 | 0.1 |
| 1.7 | **2.1** | 4.0 | 8.0 | 0.5 |
| 1.3 | **2.3** | 4.0 | 8.0 | 1.3 |
| 2.0 | **2.4** | 4 | 17 | 3.00 |
| 1.9 | **2.5** | 4 | 17 | 3.30 |
| 1.8 | **2.5** | 4 | 17 | 3.63 |
| 1.7 | **2.5** | 4 | 17 | 4.00 |
| 1.6 | **2.5** | 4 | 17 | 4.33 |


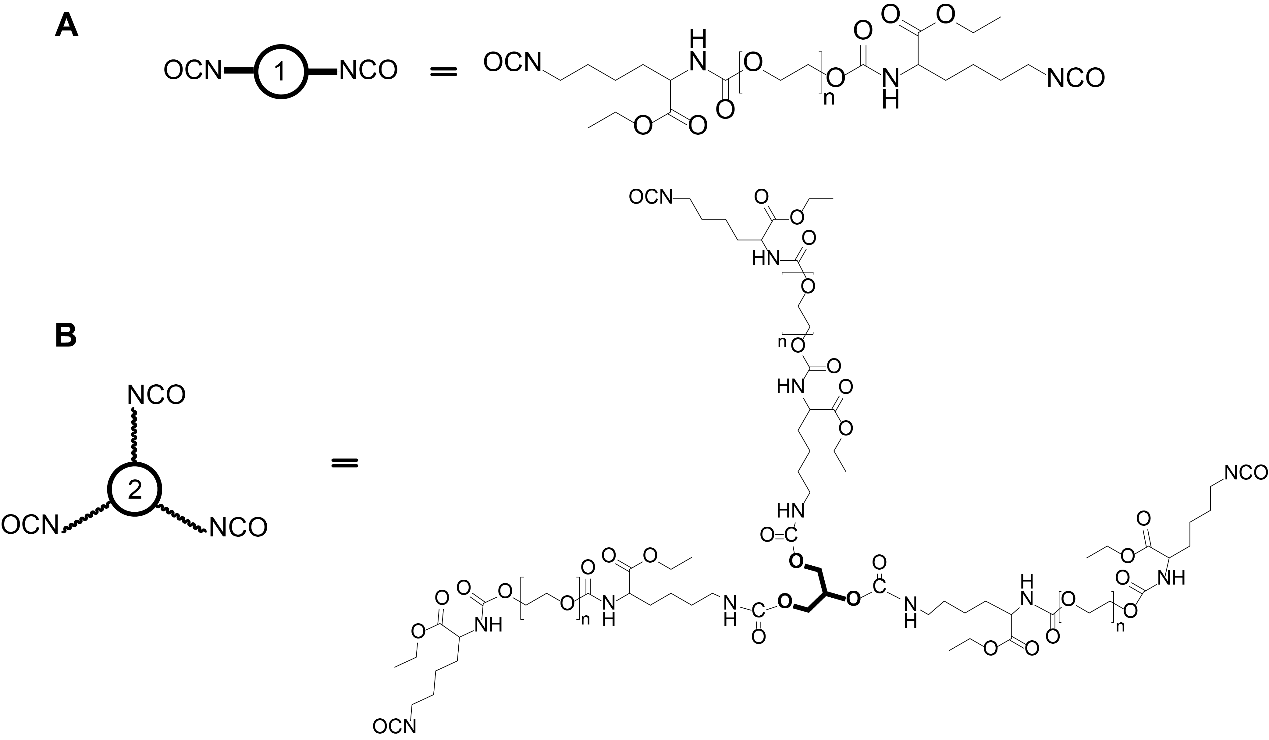


**Supplementary Figure 1.** Chemical formulas for LDI-capped PEG (**A**) and two-component polyurethane bioadhesive prepolymers (Component A) (**B**).


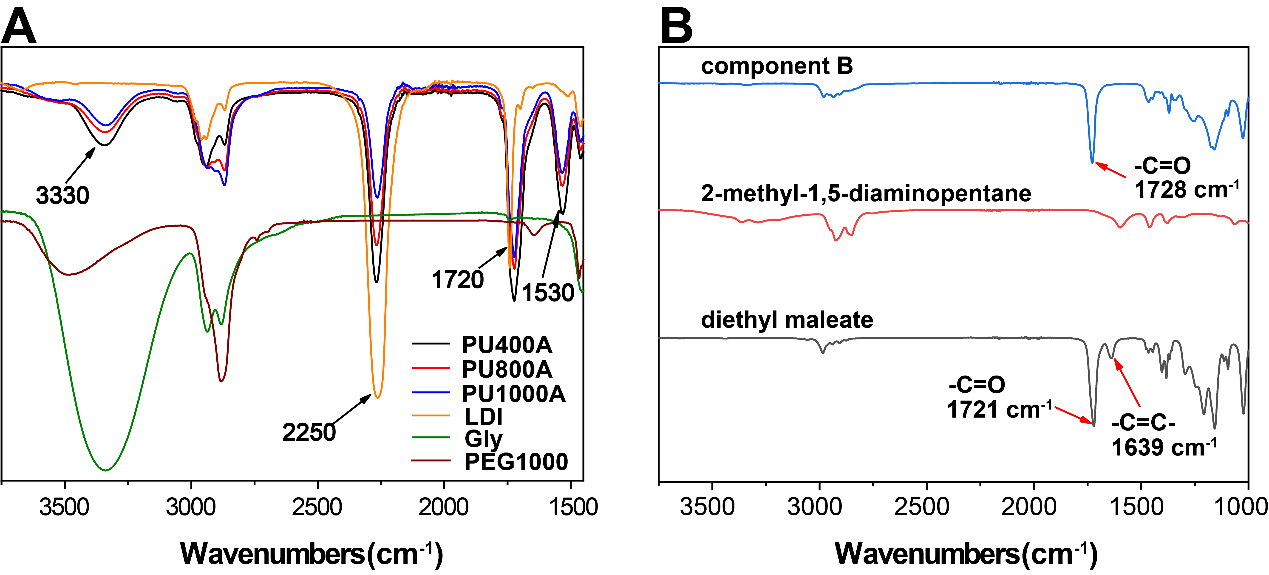


**Supplementary Figure 2.** Fourier transform infrared spectroscopy of prepolymers synthesized from PEG with different molecular chain lengths (**A**) and Component B (**B**).


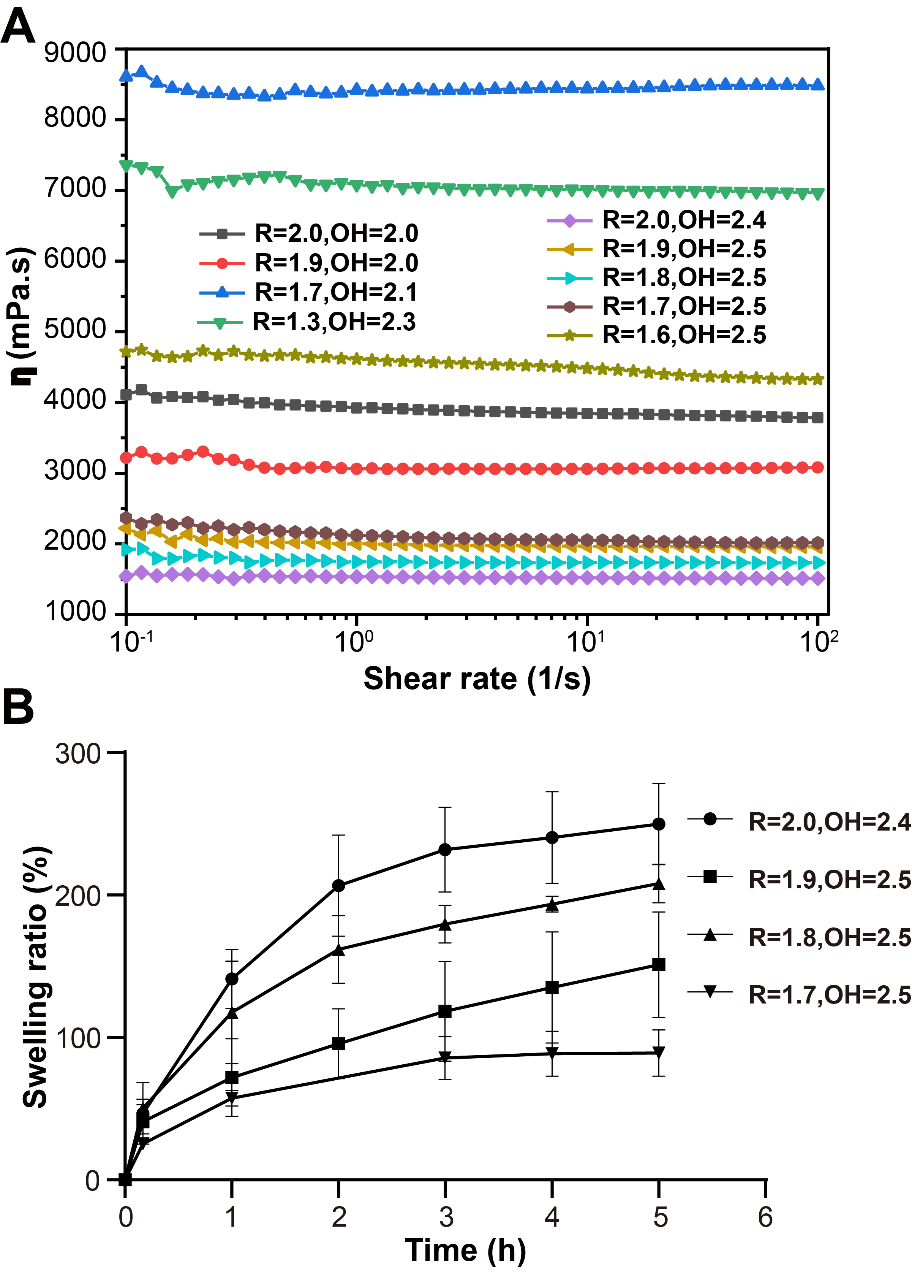


**Supplementary Figure 3.** Viscosity of polyurethane prepolymers (**A**) and swelling properties of the adhesive bulk (**B**) at varying R and OH values


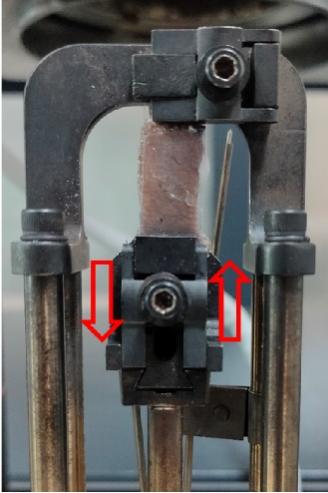


**Supplementary Figure 4.** The process of shear adhesion durability testing for the PU1000S adhesive on wet skin via a dynamic mechanical analyzer (DMA).


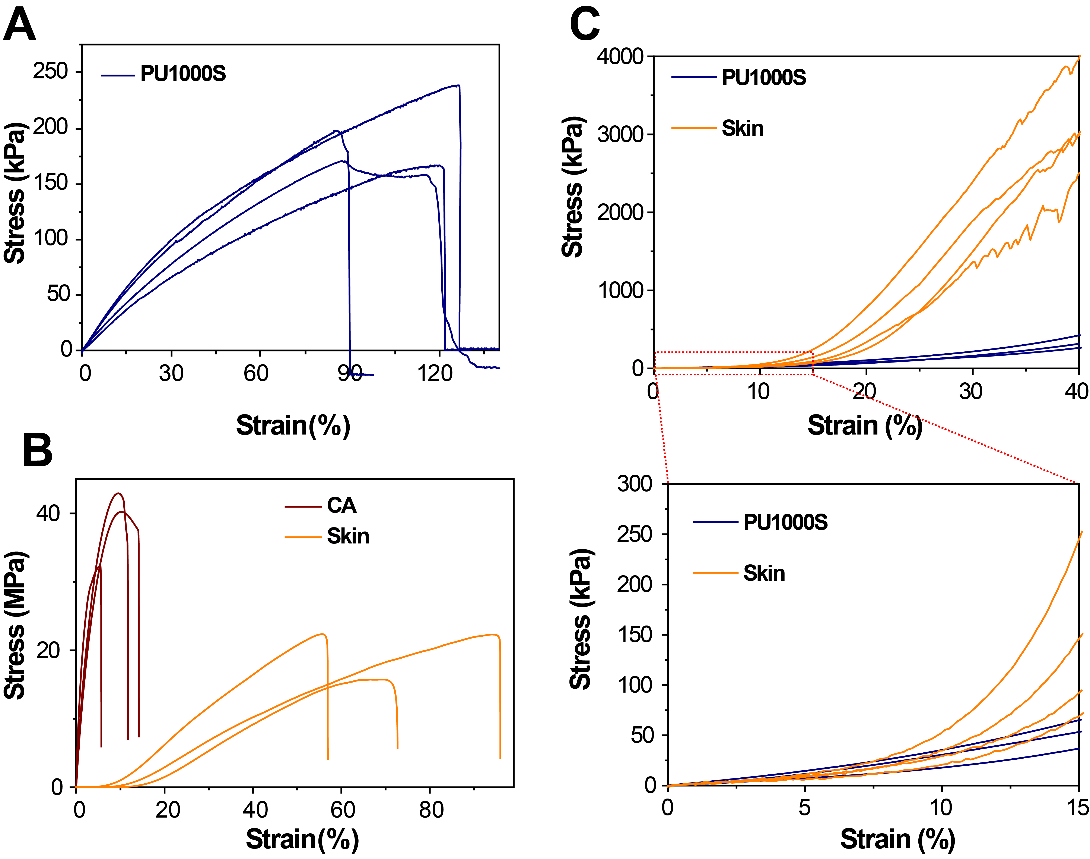


**Supplementary Figure 5.** Tensile and compression tests of PU1000S and N-butyl α-cyanoacrylate tissue adhesive (CA) adhesives and porcine skin.

Tensile stress‒strain curves of the PU1000S adhesives in the dry and swollen states (**A**). Tensile stress‒strain curves of CA adhesive and porcine skin (**B**). Compression stress‒strain curves of the PU1000S adhesive and porcine skin (**C**).


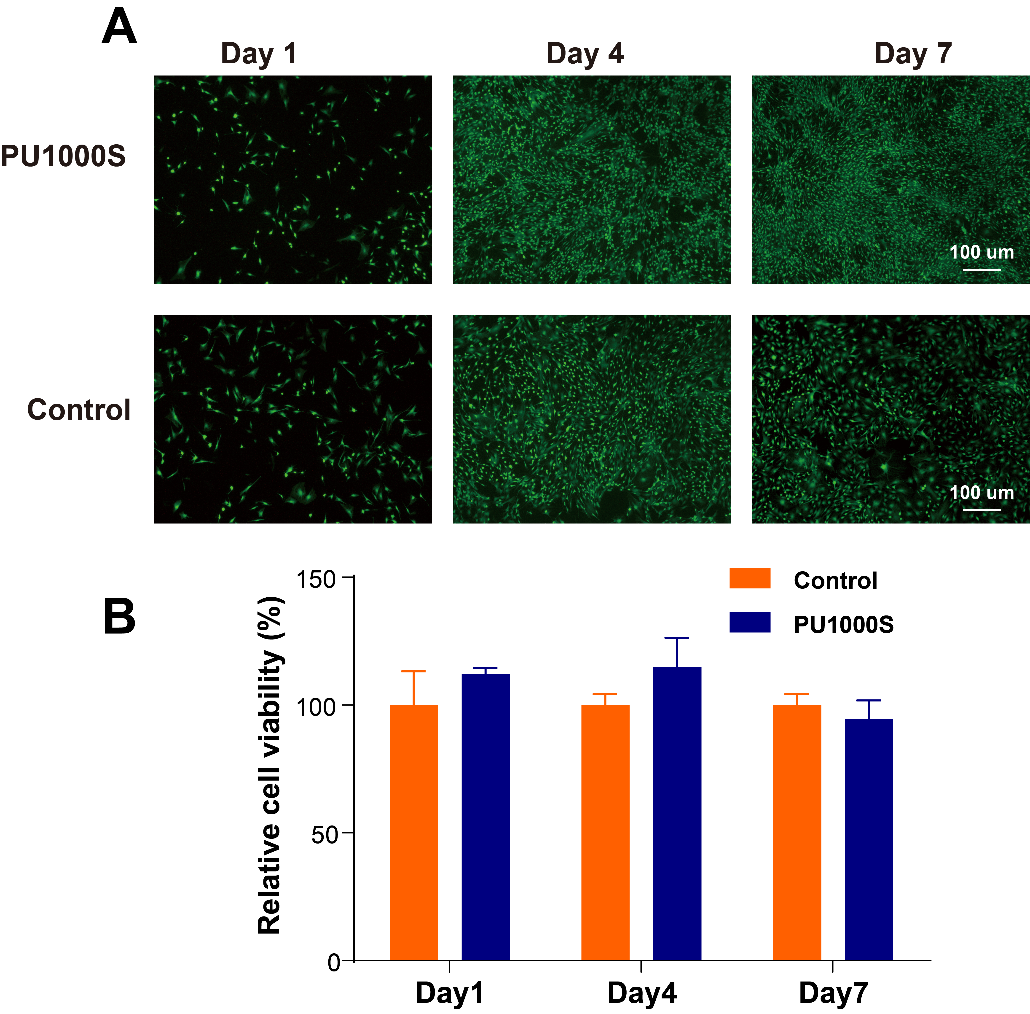


**Supplementary Figure 6**. Live/dead fluorescence images (**A**) and relative cell viability (**B**) on the 1st, 4th and 7th days for the PU1000S adhesive.


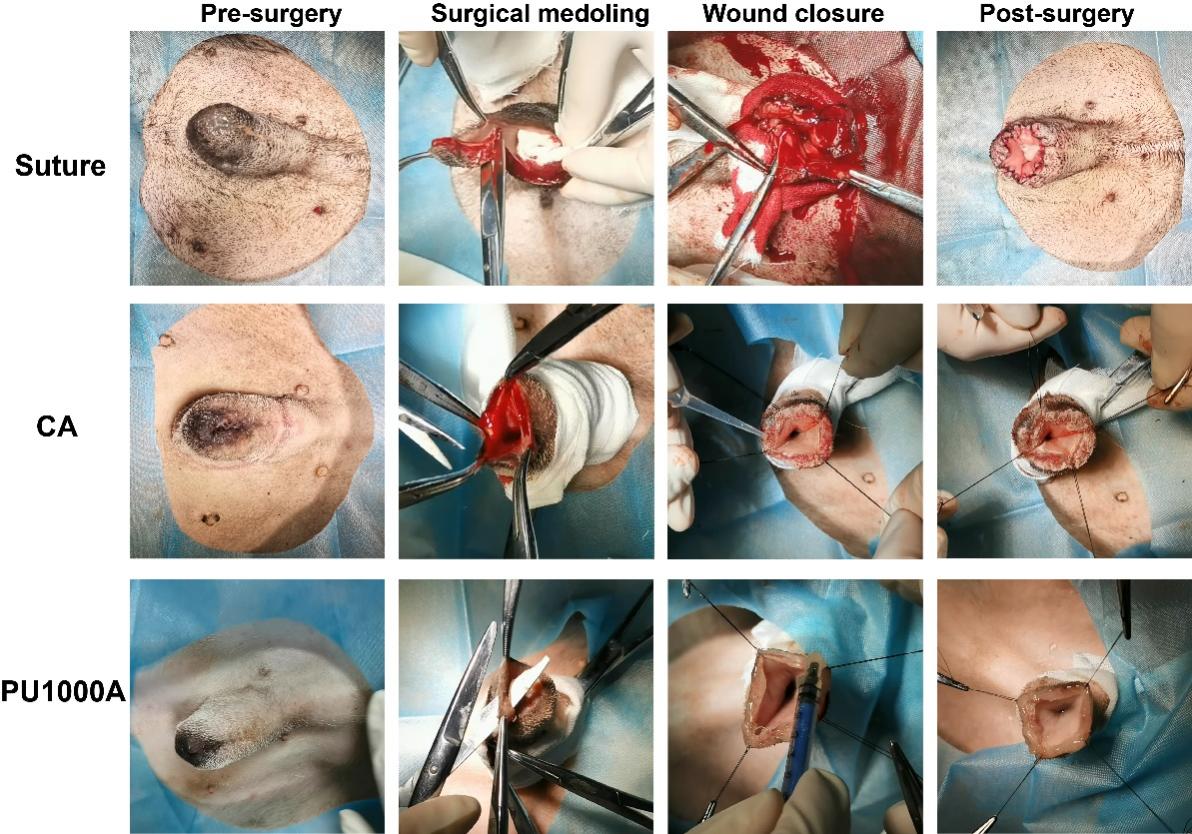


**Supplementary Figure 7**. Circumcision procedure and wound closure methods and effects in a Labrador dog model for suture, N-butyl α-cyanoacrylate tissue adhesive (CA) and PU1000S adhesive groups.
